# Tailoring the Properties of Magnetite/PLA Nanocomposites: A Composition-Dependent Study

**DOI:** 10.3390/polym17121713

**Published:** 2025-06-19

**Authors:** Mariana Martins de Melo Barbosa, Juliene Oliveira Campos de França, Quezia dos Santos Lima, Sílvia Cláudia Loureiro Dias, Carlos A. Vilca Huayhua, Fermín F. H. Aragón, José A. H. Coaquira, José Alves Dias

**Affiliations:** 1 Laboratório de Catálise, Instituto de Química, Universidade de Brasília, Campus Universitário Darcy Ribeiro, Asa Norte, Brasília 70910-900, DF, Brazil; julienechemistry@gmail.com (J.O.C.d.F.); quezia.sl198@gmail.com (Q.d.S.L.); scdias@unb.br (S.C.L.D.); 2 Laboratório de Síntese de Nanomateriais e Caracterização Magnética-Núcleo de Física Aplicada, Instituto de Física, Universidade de Brasília, Brasília 70910-900, DF, Brazil; cavilcahu@gmail.com (C.A.V.H.); ffharagon@gmail.com (F.F.H.A.); 3 Departamento de Ciencias, Sección Física, Pontificia Universidad Católica del Perú, Av. Universitaria 1801, Lima 15088, Peru

**Keywords:** Poly(lactic acid)-PLA, magnetite, magnetic nanoparticles (MNP), nanocomposites MNP/PLA, PLA biocompatible polymer

## Abstract

This study focused on composites of magnetite magnetic nanoparticles (MNP) and poly(lactic acid) (PLA) prepared via sonochemical synthesis. The evaluation of MNP loadings (2, 5, 10, 15, and 20 wt.%) provided insights into the structural and reactivity properties of the materials. Methods used included XRD, FT-IR and Raman spectroscopy, SEM and TEM microscopy, textural and thermal analysis (TG and DTA), and magnetic property measurements. The agreement between theoretical and experimental MNP loadings was good. XRD patterns showed predominantly MNP and semicrystalline phases, with a minor maghemite phase detected by FT-Raman and magnetic measurements. FT-IR analysis revealed interactions between MNP and PLA, confirmed by thermal analysis showing higher transition temperatures for the composites (145 °C) compared to pure PLA (139 °C). FT-Raman spectra also indicated that PLA helps prevent iron oxide oxidation, enhancing nanoparticle stability. SEM and TEM micrographs showed well-dispersed, spherical nanoparticles with minimal agglomeration, dependent on MNP loading. The nanocomposites exhibited low N_2_ adsorption, resulting in low surface area (~2.1 m^2^/g) and porosity (~0.03 cm^3^/g). Magnetic analysis indicated that in the 2MNP/PLA sample, MNP were in a superparamagnetic-like regime at 300 K, suggesting good dispersion of 2 wt.% MNP in the PLA matrix.

## 1. Introduction

Poly(lactic acid), or polylactide (PLA), is one of the main bioplastics produced in the world [1]. In 2020, it was reported that 40% of the bioplastics market consisted of common bio-based plastics such as polylactic acid (PLA), polyhydroxyalkanoates (PHA), and thermoplastic starch (TPS) [2]. PLA is used in a variety of applications, including the packaging industry, automotive industry, air filtration, and the biomedical field [3,4]. Since the work of Kulkarni and coworkers [5], who demonstrated the biocompatibility of PLA, the polymer has been extensively studied for use in biomedicine. Moreover, the fact that its degradation products (water, carbon dioxide, and lactic acid) are considered non-toxic and safe makes it even more appealing for biomedical applications [4]. In many fields, however, the addition of other materials to PLA can enhance certain properties or impart new characteristics to better suit specific purposes. Magnetite is one such material that has been studied for the synthesis of magnetic PLA composites [3,6].

Magnetite magnetic nanoparticles (MNPs) are superparamagnetic, nontoxic, and biocompatible—properties that make them highly desirable in drug delivery systems [7,8]. When incorporated into drug carriers, magnetite enables the magnetic targeting of pharmaceuticals to diseased tissues and facilitates the potential use of magnetic hyperthermia in cancer treatments [7]. Additionally, the inclusion of magnetite in PLA-based bone scaffolds or screws enhances radiopacity and provides strong contrast in magnetic resonance imaging (MRI) scans [9,10]. Magnetic polymeric composites are also being explored for use in shape-memory materials within the emerging field of 4D printing [11]. Furthermore, adding magnetite nanoparticles to PLA air filters has been shown to improve their filtration performance [12].

The interactions between magnetite and PLA in a composite lead to changes in the characteristics of both materials. The addition of magnetite to a PLA matrix can decrease the thermal stability of the polymer and its blends [13,14]. However, this effect must be balanced against the desired saturation magnetization. While lower magnetite loading can result in reduced magnetization [15], particle–particle interactions may also influence magnetization, sometimes leading to higher saturation magnetization despite lower magnetite content [16]. Regarding the effect of inorganic additives on the crystallinity of the polymer, the results reported in the literature vary. In a study conducted by Bakr and coworkers [17], the addition of magnetic nanocellulose to PLA had no effect on the polymer’s crystallinity; however, when magnetite was added to a PLA and nanocellulose copolymer, the crystallinity of the copolymer decreased. In contrast, a study by Pekdemir and collaborators [18] demonstrated that the addition of magnetite to a PLA/PEG blend increased the degree of crystallinity of the blend. The porosity of magnetite/PLA composites is not well documented in the literature, with most studies assessing porosity through microscopic techniques or weight measurements [19,20].

Several methods have been employed to synthesize composites of PLA and magnetite, with many of these processes tailored for specific applications [14,21,22,23]. The use of additional molecules to functionalize the magnetite nanoparticle is common, considering the low compatibility between magnetite and PLA. Studies have been conducted on the formation of magnetite and PLA composites without functionalization, including a recent one from our group [24,25]. In that research, we used the sonochemical method to enhance dispersion of MNP into PLA without the use of surfactants or organic solvents [25].

The present study aims to advance the sonochemical synthesis of PLA composites with varying magnetite loadings. The structure and properties of the resulting composites were thoroughly evaluated using several methods, including powder X-ray diffraction (XRD), Fourier-transform infrared and Raman spectroscopies (FT-IR and FT-Raman), scanning and transmission electron microscopy (SEM and TEM), analysis of textural properties by N_2_ physisorption, thermal analysis, and magnetic measurements. Porosity and magnetism, in particular, are relatively novel properties being explored in these materials.

## 2. Experimental Procedures

### 2.1. PLA Synthesis

PLA synthesis was carried out through the direct polycondensation of D,L-lactic acid, using a protonic silica-alumina catalyst (12 wt.% Al_2_O_3_, Sigma-Aldrich, Saint Louis, MO, USA). The process occurred in two steps: pre-polymerization and catalytic direct polycondensation.

In the first step, 15 mL of D,L-lactic acid (85%, Vetec, São Paulo, Brazil) was added to a three-necked round-bottom flask equipped with a condenser to remove water. The system was maintained at 160 °C under a nitrogen gas flow (industrial grade, 99.99%, White Martins, Brazil) and magnetic stirring at 340 rpm for 4 h. In the second step, 0.02 g of the catalyst was added to the system, and the condenser and gas flow were removed. A vacuum hose was then attached, and the system was sealed hermetically. The temperature was increased to 180 °C, and the reaction was continued for 15 h. Afterward, the polymer was cooled to ambient temperature and dissolved in 10 mL of chloroform (99.8%, Cromoline, Brazil). The catalyst was removed by centrifugation, and the polymer was precipitated with methanol (99.8%, J.T. Baker, London, UK). The methanol and chloroform were then removed via rotary evaporation, and the polymer was ground into a fine powder using an agate mortar and pestle. Detailed information on the synthesis can be found in the literature [26,27].

### 2.2. MNP/PLA Composite Syntheses

As indicated initially, PLA was prepared following the established methodology extensively documented in the literature [25,26,27]. Then, the composites were synthesized as previously reported [25], according to the chemical reaction:

$$6{Fe}^{2+}+O_{2}+12{OH}^{-}\to 2{Fe}_{3}O_{4}+6H_{2}O$$

Thus, for the 5 wt.% magnetite to PLA composite (5MNP/PLA), 0.10 g of ferrous sulfate (FeSO_4_·7H_2_O, Merck, USA) and 0.95 g of PLA, previously synthesized, were added to a round bottom flask with 100 mL of Milli-Q water (Merck Millipore, model Direct 8, Guyancourt, France). The system was placed in an ultrasonic bath (SolidSteel, model SSBuc-6L, Brazil, operating at 40 kHz) and 4 mL of ammonium hydroxide (27%, Alphatec, Sao Paulo, Brazil) was added dropwise. The reaction was maintained for 1 h under ultrasonic radiation and the resulting precipitate was separated with a neodymium magnet and washed with Milli-Q water. The composite was dried in an oven at 50 °C for 3 h. This procedure was repeated for composites (xMNP/PLA) with x = 2.5, 10, 15, and 20 wt.% of magnetite to PLA, i.e., forming 2MNP/PLA, 10MNP/PLA, 15MNP/PLA, and 20MNP/PLA, respectively. Pure magnetite was synthesized by the same method without the addition of the polymer as a reference. The average yield of these syntheses was about 65–70%, which agreed with our previous procedure [25].

### 2.3. Characterization Methods

#### 2.3.1. Powder X-Ray Diffraction (XRD)

Powder diffraction patterns were obtained in a D8 Focus diffractometer, from Bruker (Rheinstetten, Germany) with radiation of Cu-Kα of 0.15418 nm at 40 kV and 30 mA, in the range of 2θ = 10–70° with increments at 0.02 at 0.5° min^−1^.

#### 2.3.2. Fourier-Transform Infrared Spectroscopy (FT-IR)

FT-IR spectra were obtained using a Nicolet 6700 spectrometer, from Thermo Fisher Scientific (Waltham, MA, USA), with 256 scans, a resolution of 4 cm^−1^, and transmittance mode, using KBr pellets (1 wt.%).

#### 2.3.3. Fourier-Transform Raman Spectroscopy (FT-Raman)

Raman spectra were acquired using a LabRAM HR Evolution spectrometer from Horiba (Montpellier, France) under ambient conditions (25 °C). A 795 nm laser was employed at different power levels: 90 mW for the PLA sample, 9 mW for the magnetite sample, and 4.5 mW for the composite samples. Each spectrum was recorded over 64 acquisitions with a spectral resolution of 2 cm^−1^.

#### 2.3.4. Microscopy Analyses

The SEM images were obtained on a JSM-6610 microscope, from Jeol (Tokyo, Japan). TEM images were acquired with a JEM 2100 transmission electron microscope, from Jeol (Japan) operating at 200 kV. The powder was dispersed in ethyl alcohol using an ultrasonic bath, followed by placing it on a copper grid. The SEM microscope was equipped with energy dispersive X-ray spectroscopy (EDX) from Thermo Scientific NSS Spectral Imaging. Morphological evaluation and nanoparticle size estimation were performed using images obtained by transmission electron microscopy (TEM). Particle diameters were measured with ImageJ software (TA-60WS Thermal Analysis Workstation software, version 2.21.), based on the scale bars provided in the micrographs. To ensure statistical relevance, at least 150 particles were analyzed for the 2MNP/PLA sample, and more than 200 particles were measured for the other composites. Particle size distribution was plotted as histograms and fitted to a log-normal distribution using OriginPro 2024.

#### 2.3.5. Magnetic Analysis

The hysteresis, zero-field cooled (ZFC) and field cooled (FC) curves were obtained on a vibrating sample magnetometer (SQUID) from Quantum Design with maximum magnetic field of 70 kOe in the range of temperature of 5 K to 300 K.

#### 2.3.6. Thermal Analysis (TG/DTG and DTA)

Thermogravimetric (TG), derivative thermogravimetric (DTG) and differential thermal analyses (DTA) were performed on a TG analyzer, model DTG-60H from Shimadzu (Japan) with a heating rate of 10 °C/min in the range of ambient temperature (25 °C) to 600 °C and a flux of 50 mL/min of synthetic air (±20% O_2_ and ±80% N_2_, 99.999% purity) from White Martins (Brazil). It was used TA-60WS Thermal Analysis Workstation software, version 2.21. Approximately 10 mg of the sample was used in a platinum crucible.

#### 2.3.7. Textural Properties

Textural properties of the materials were obtained on a Micromeritics ASAP 2020C (Accelerated Surface Area and Porosimetry System, USA) by N_2_ adsorption/desorption at −196 °C. Degasification was carried out under low pressure (target of 50 μmHg) at temperature of 50 °C for 4 h before the physisorption experiment. In total, 0.5 to 1.0 g of each powdered solid sample was used.

## 3. Results and Discussion

### 3.1. Characterization of PLA and MNP Materials

The synthesized PLA polymer was characterized using ^1^H and ^13^C NMR spectroscopy, as well as X-ray diffraction (XRD), while the structure of the magnetite magnetic nanoparticles (MNPs) was confirmed by XRD.

The ^1^H NMR spectrum of the polymer (Figure S1a) shows characteristic signals of PLA at 1.5 and 1.45 ppm, corresponding to the protons of the methyl groups (–CH_3_) in the repeating units and in the group linked to the terminal –OH, respectively [28]. Notably, a quartet at 4.5 ppm, characteristic of methine protons in the polymer chain, indicates that the polymer is predominantly composed of a single isomer [29]. According to our previous research, the polymer contains an excess of the L-isomer [25]. The ^13^C NMR spectrum (Figure S1b) is characteristic of poly(L-lactic acid) (PLLA), with signals at 16 ppm for the methyl carbon, 69 ppm for the methine carbon (–CH), and 169 ppm for the carbonyl carbon [30].

The XRD pattern of the polymer (Figure S2) corresponds to a semi-crystalline PLA homopolymer, showing peaks at 2θ = 14.9° (010), 16.8° (110/200), 19.2° (203), and 22.5° (015) [31], along with a subtle peak at 2θ = 12.6°, which is characteristic of stereocomplex (sc) PLA [32,33]. The XRD pattern of magnetite (Figure S2) displays features typical of the inverse spinel structure of this iron oxide [25,34]. Additional details about these materials are available in previous studies by our group [25,26,27].

### 3.2. Elemental Analysis of MNP/PLA Nanocomposites

The total thermal degradation of the PLA polymer at 600 °C was used to determine the inorganic content of the materials through thermogravimetric analysis (TGA). At this temperature, the remaining residue consists solely of Fe_2_O_3_, allowing the calculation of the original magnetite content in the composite (Table 1). The difference between the theoretical (2.5, 5, 10, 15, and 20 wt.%) and experimental values was found to be less than 2%. Therefore, the samples were labeled according to the original theoretical values.

### 3.3. XRD of xMNP/PLA Nanocomposites

**Figure 1:**
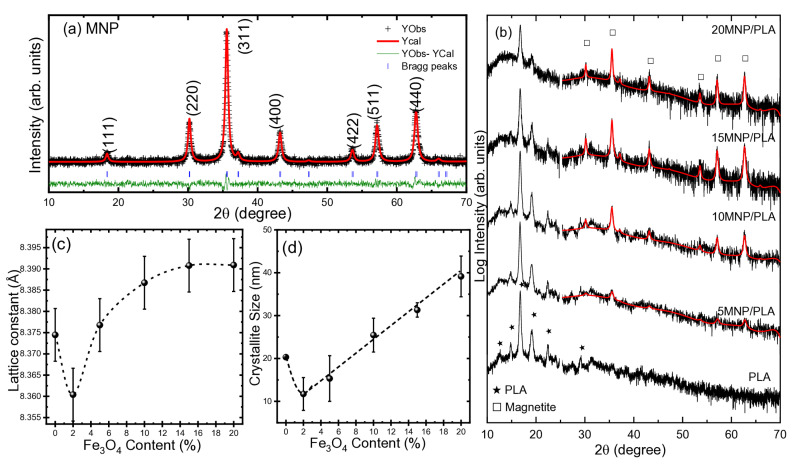
X-ray diffraction (XRD) patterns of (**a**) pure magnetite nanoparticles (MNP) and (**b**) MNP/PLA composites with varying MNP content (5, 10, 15, and 20 wt.%) as well as pure PLA. In panel (**a**), experimental data (Y_Obs_, black crosses) are analyzed using Rietveld refinement (Y_Cal_, red line), with the difference plot (Y_Obs_ − Y_Cal_, green line) and Bragg reflection positions (blue ticks) shown. In panel (**b**), the peaks corresponding to magnetite are marked with squares (□), and PLA are marked with stars (★); (**c**,**d**) variation in the lattice constant and crystallite size with increasing magnetite content in the MNP/PLA composites. The dashed lines are provided only as a guide to the eyes.

XRD analysis revealed the presence of both magnetite and PLA phases. The XRD patterns (Figure 1a) show the magnetite phase clearly identified based on the diffraction peaks at 2θ = 18.5° (111), 30.2° (220), 35.5° (311), 43.2° (400), 53.5° (422), 57.1° (511), and 62.7° (440). These reflections are characteristic of the inverse spinel structure of magnetite [34,35], and no additional phases were detected. To provide more detailed information, Rietveld refinement was carried out in the 2θ region up to 25°, which includes the main diffraction peaks characteristic of magnetite. As shown in Figure 1a, the points represent the experimental data, while the solid line corresponds to the calculated pattern. The obtained lattice constant was 8.3745 ± 0.0002 Å, which is in good agreement with values reported in the literature [36]. It should be noted that, since maghemite and magnetite both crystallize in a cubic phase, their diffraction patterns are very similar and can be difficult to distinguish in this case. The average crystallite size of the pure MNPs was estimated to be 20 ±1 nm, which is determined using the Scherrer equation. In addition, for pure PLA, the presence of semi-crystalline homopolymer PLA is characterized by peaks at 2θ = 14.9° (010), 16.8° (110/200), 19.2° (203), and 22.5° (015) [10,21]. According to previous research, the polymer contains an excess of the L-isomer [25]. The XRD patterns also show the presence of stereocomplex crystals (sc) of PLA, identified by the peak at 2θ = 12.6° [14,22]. These peaks are observed in nearly all the composites, except for the 20MNP/PLA (Figure 1b), in which the lower intensity peaks are not visible. Therefore, a higher concentration of magnetite may affect the crystallinity of the polymer.

The structural analysis revealed a clear trend of increasing lattice parameter and crystallite size with higher magnetite content in the MNP/PLA composites, as it is displayed in Figure 1c and Figure 1d, respectively. The lattice constant exhibited a slight increase as the concentration of magnetite increased, which could be attributed to the reduction in interfacial strain between the magnetite nanoparticles and the PLA matrix. At lower concentrations, the polymer may exert a mechanical enclosing effect on the nanoparticles, slightly distorting their crystal structure. As the magnetite content rises, particle agglomeration and reduced interaction with the surrounding PLA allow the crystal lattice to relax, approaching a more crystalline value of magnetite (8.396 Å). Furthermore, higher magnetite loading may suppress surface oxidation to maghemite (8.34 Å), known to have a smaller lattice parameter, contributing to the observed increase. Similarly, the crystallite size, obtained from Rietveld refinement of the XRD patterns, showed a positive correlation with the magnetite content. This increase is likely due to reduced spatial confinement by the PLA matrix at higher loadings, which facilitates nanoparticle coalescence and grain growth. At low magnetite concentrations, the nanoparticles are better dispersed within the polymer, and their growth is restricted by spatial constrains from the surrounding PLA chains. In contrast, higher concentrations favor the formation of larger crystalline domains due to aggregation and percolation effects, leading to broader particle size distributions and enhanced total crystallinity.

### 3.4. FT-IR of xMNP/PLA Nanocomposites

**Figure 2:**
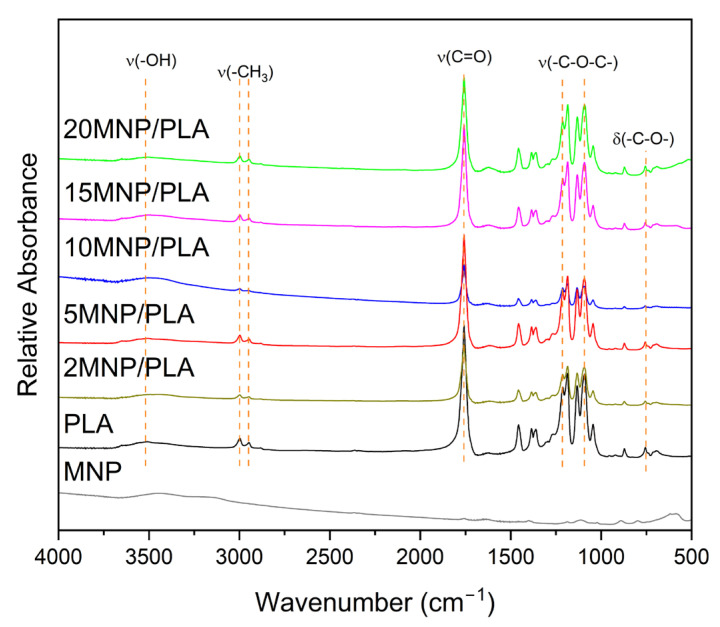
Infrared spectra of PLA, MNP, and the composite samples.

The infrared spectra (Figure 2) of the composites and the polymer exhibit characteristic bands of PLA. The broad band at 3508 cm^−1^ in the pure polymer corresponds to the stretching of the -OH group. This band appears at lower wavenumbers in the 10MNP/PLA (3495 cm^−1^) and 15MNP/PLA (3499 cm^−1^) composites, indicating an interaction between the polymer and magnetite. The bands at 2998 and 2959 cm^−1^ represent the symmetric and asymmetric stretching of the -CH_3_ bond. The sharp band at 1759 cm^−1^ is associated with the stretching of the C=O bond. The asymmetric and symmetric stretching of the -C-O-C- bond are represented by the bands at 1215 cm^−1^ and 1092 cm^−1^, respectively. The band at 752 cm^−1^ corresponds to the bending of the -C-O- bond. Magnetite, on the other hand, shows two characteristic bands at 600–550 cm^−1^ and 451 cm^−1^, related to the stretching of the Fe-O bond [37].

**Figure 3:**
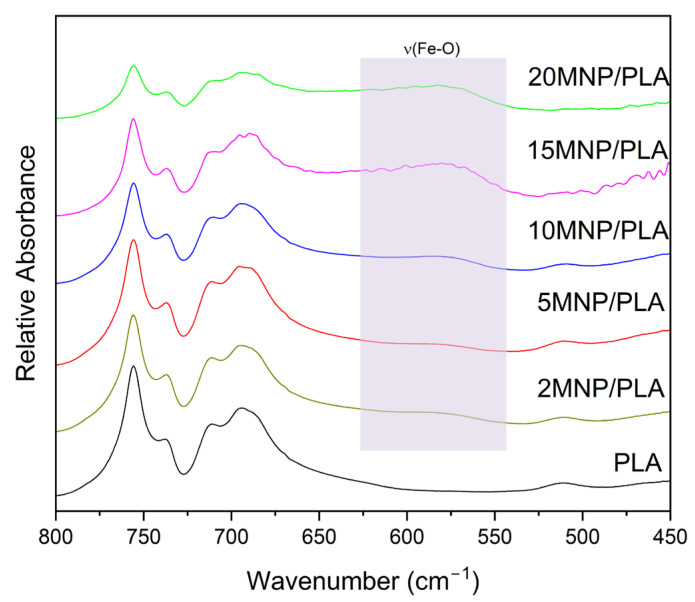
Infrared spectra of PLA and composites highlighting the Fe-O bond band.

Figure 2 shows faint bands, prompting further studies with a higher concentration of composites in the KBr pellet (10 wt.%). Thus, Figure 3 clearly shows the broad bands related to Fe-O in the composites, confirming the presence of magnetite.

### 3.5. Thermal Stability of xMNP/PLA Nanocomposites

**Figure 4:**
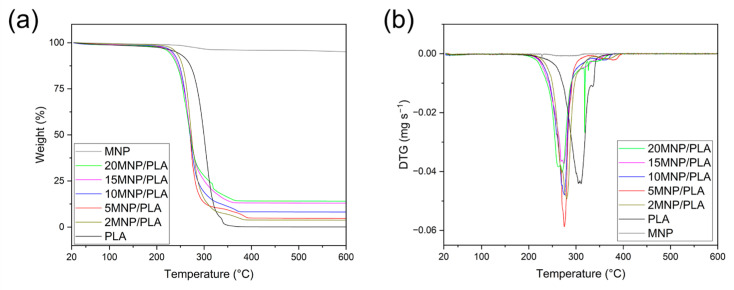
TG (**a**) and DTG (**b**) curves of PLA, MNP, and the composite samples under synthetic air.

Thermogravimetric analysis (TG) of the MNP (Figure 4a) shows the desorption of water molecules and hydroxyl groups from the magnetite surface. Furthermore, the polymer in this temperature range was completely decomposed, as indicated by the approximate near-zero residual mass at the maximum temperature of 600 °C. The inorganic content of the composites was estimated based on the residual mass after the complete degradation of PLA, as discussed in the elemental analysis section. The temperature of maximum degradation (T_d_) for PLA was 308 °C, as shown in Figure 4b.

The composites, however, exhibit two distinct regions of mass loss: one at a temperature lower than the polymer’s T_d_ (T_1_), and another above T_d_ (T_2_). The loss of thermal stability in PLA could be attributed to the presence of iron and the addition of a base (ammonia) in the synthesis process, both of which can accelerate PLA hydrolysis and degradation [38]. On the other hand, the increased thermal stability may result from electrostatic or chemical interactions between the polymer and magnetite [25,39], as well as the immobilization of the polymer chains and their degradation products due to these interactions [40]. The values for T_1_ and T_2_ are provided in Table 2. T_1_ values for all composites fall within similar ranges, except for the 20MNP/PLA composite, which shows a slightly lower temperature, and the 2MNP/PLA composite, which shows the least loss of thermal stability. Regarding T_2_, composites with higher concentrations of MNP display a lower increase in thermal stability, while the 5MNP/PLA composite shows the highest gain at a temperature of 371 °C.

**Figure 5:**
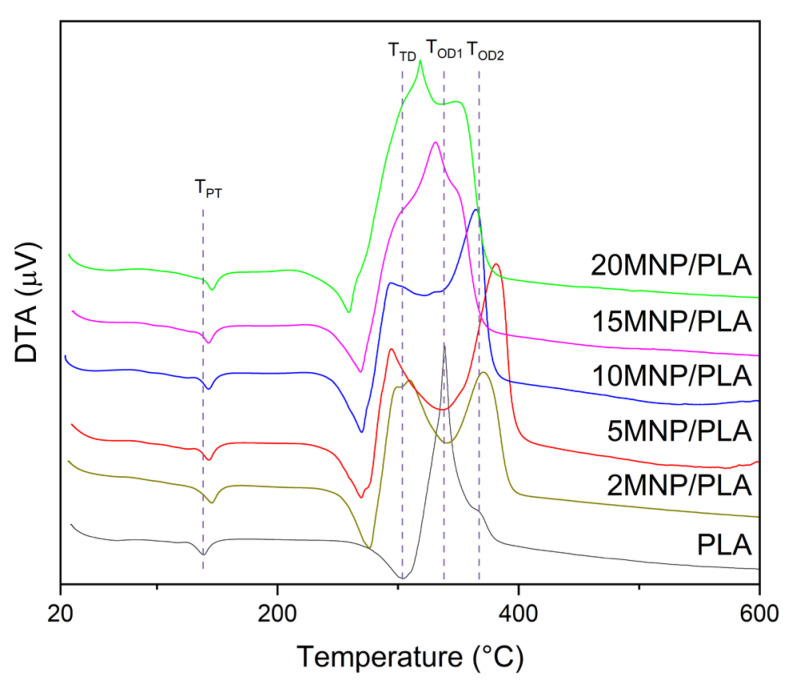
DTA curves of PLA and the composite samples under synthetic air.

Differential Thermal Analysis (DTA) was also performed (Figure 5). All samples show two endothermic peaks below or at 300 °C and two exothermic peaks above 300 °C. The first endothermic peak indicates a phase transition of the polymer, i.e., melting, while the second corresponds to thermal degradation. The two exothermic peaks are associated with the thermo-oxidative degradation of the polymer [41].

The phase transition temperature (T_PT_) of the composites is higher than that of the pure polymer (139 °C), with the highest T_PT_ observed in a composite sample at 145 °C. This increase is likely due to interactions between the polymer and magnetite [40]. Additionally, previous studies have documented how the addition of magnetite affects the fluid dynamics of PLA, requiring higher temperatures for extrusion to form filaments [42]. The thermal degradation temperature (T_TD_) of all composites is lower than that of the pure polymer. While pure PLA exhibits a T_TD_ of 305 °C, the T_TD_ of the composites falls within the range of 270 to 276 °C, except for the 20MNP/PLA composite, which has a T_TD_ of 259 °C. This is consistent with the aforementioned loss of thermal stability in the polymer due to the addition of magnetite.

Regarding the oxidative degradation temperatures (T_OD1_ and T_OD2_), the first peak (T_OD1_) in all composites occurs at lower temperatures than in pure polymer. However, unlike other thermal behaviors, composites with higher concentrations of magnetite exhibit a T_OD1_ higher than those with lower concentrations. For T_OD2_, the 5MNP/PLA composite shows a higher temperature (381 °C) compared to pure PLA (366 °C). In contrast, the 15MNP/PLA and 20MNP/PLA composites show a T_OD2_ lower than that of the pure polymer. All temperature values are summarized in Table 3.

The degradation of PLA occurs through various mechanisms [38]. One of the primary mechanisms, observed in thermogravimetric analysis, is the generation of macroradicals when heated [43]. The thermo-oxidative degradation process then leads to the formation of new free radicals from chain scission, continuing until complete polymer degradation [38]. As previously mentioned, iron can act as a catalyst in the degradation process, which explains the lower T_TD_ and T_OD1_ temperatures in all composites. However, the increase in T_OD1_ with rising nanoparticle concentration is unexpected. This suggests that magnetite not only acts as a catalyst but also serves as a barrier, limiting the diffusion of oxygen and heat into the polymer matrix. As a result, a higher concentration of magnetite strengthens this barrier effect. A similar trend has been observed with nanoclay [40]. The higher T_OD1_ for the 2MNP/PLA composite compared to the 5MNP/PLA composite may occur because the lower concentration of magnetite is sufficient to disrupt the length of the polymer chains, but not enough to serve as an effective catalyst for the degradation of PLA. Regarding the stability gain in the 5MNP/PLA for T_OD2_, the nanoparticles may trap the free radicals generated, thereby slowing PLA degradation [44]. However, for the 15MNP/PLA and 20MNP/PLA composites, catalytic effects appear to dominate in this temperature range.

### 3.6. FT-Raman of xMNP/PLA Nanocomposites

**Figure 6:**
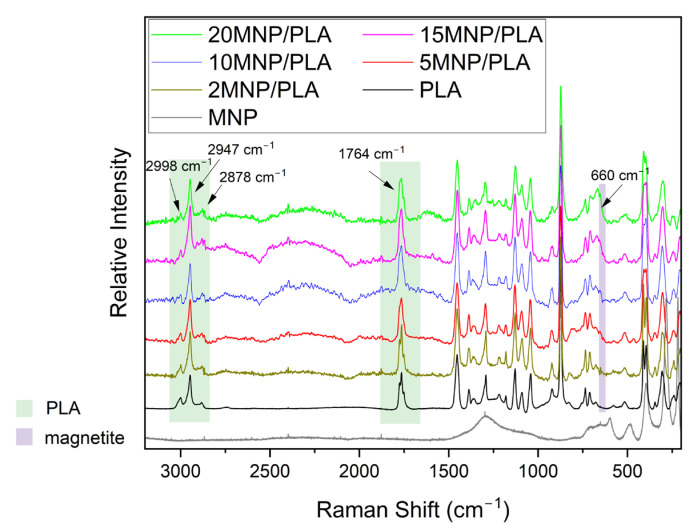
Raman spectra of samples obtained using a 795 nm laser with varying power: 90 mW for the PLA sample, 9 mW for the magnetite sample, and 4.5 mW for the composite samples.

**Figure 7:**
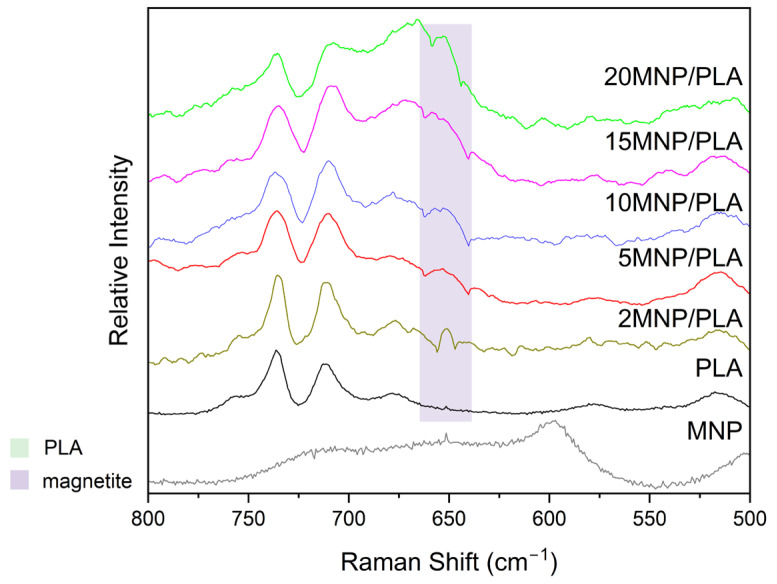
Raman spectra of samples in the 500–800 cm^−1^ range, obtained using a 795 nm laser with varying power: 90 mW for the PLA sample, 9 mW for the magnetite sample, and 4.5 mW for the composite samples.

The Raman spectrum (Figure 6) of pure magnetite (Fe_3_O_4_) exhibits a clear hematite (Fe_2_O_3_) pattern, resulting from high laser power, with peaks at 225 and 498 cm^−1^ corresponding to the A_1g_ modes, and bands at 247, 290, and 400 cm^−1^ corresponding to the E_g_ modes [45,46]. The spectra of the composites, on the other hand, display characteristic PLA bands including three bands around 3000 cm^−1^, which are indicative of -CH and -CH_3_ group stretching, as well as a band at 1764 cm^−1^, indicative of C=O stretching [30]. Also, the composite spectra also feature a characteristic magnetite band. As shown in Figure 7, the composites display a distinct band around 650 cm^−1^, characteristic of the A_1g_ mode of magnetite [45,47,48]. The intensity of these bands increases with higher magnetite concentrations. The broadening of the 650 cm^−1^ band at higher concentrations may also be attributed to the presence of maghemite (γ-Fe_2_O_3_), which typically broadens the magnetite band [49]. Since magnetite is easily oxidized to hematite at laser powers above 1 mW [50,51], it can be inferred that the polymer coats the nanoparticles, thereby protecting the iron oxide from oxidation and improving nanoparticle stability.

### 3.7. Micrography Analyses of xMNP/PLA Nanocomposites

#### 3.7.1. SEM Images

**Figure 8:**
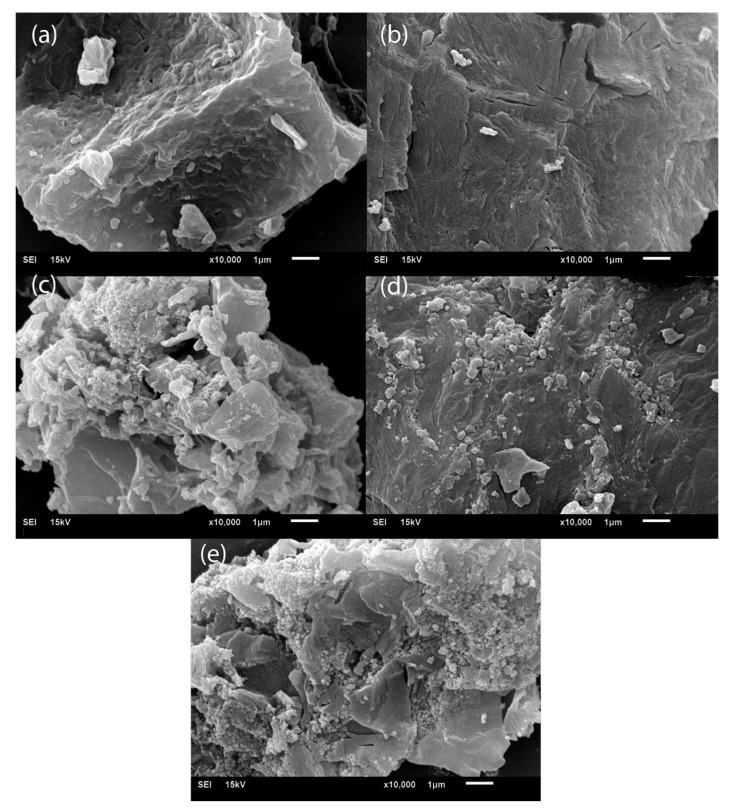
SEM micrographs of (**a**) 2MNP/PLA, (**b**) 5MNP/PLA, (**c**) 10MNP/PLA, (**d**) 15MNP/PLA, and (**e**) 20MNP/PLA composites.

The SEM micrographs of the composites (Figure 8) illustrate the dispersion of magnetite within the PLA matrix. It is evident that a higher concentration of magnetite leads to increased aggregation of the nanoparticles in the polymer. The 20MNP/PLA composite (Figure 8e) shows more agglomeration on the PLA block, covering a larger portion of the polymer, similar to the 18 wt.% composite from our previous study [25]. In that study, the composite with a higher magnetite concentration (33 wt.%) exhibited larger magnetite agglomerates on the polymer block compared to the lower concentration composite. This suggests that a higher magnetite concentration results in poorer dispersion of magnetite in the PLA matrix. The EDX elemental analysis (Figure S3) further confirms the presence of magnetite, with iron peaks detected in all composites. The intensity of these peaks increases in proportion to the magnetite concentration.

#### 3.7.2. TEM Images

**Figure 9:**
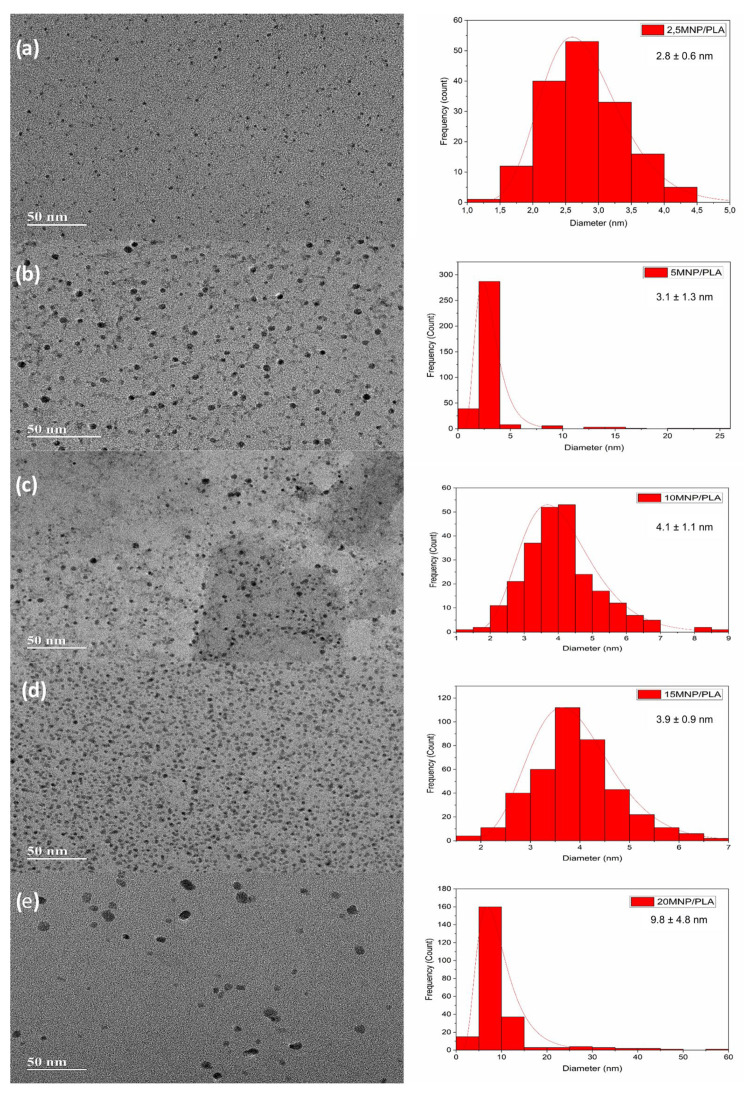
TEM micrographs (scale bar: 50 nm) of the nanocomposite samples: (**a**) 2MNP/PLA, (**b**) 5MNP/PLA, (**c**) 10MNP/PLA, (**d**) 15MNP/PLA, and (**e**) 20MNP/PLA, along with the corresponding particle size distribution histograms. The histograms display the average particle size and the associated standard deviation.

Transmission electron microscopy (TEM) analyses indicate that all synthesized composite materials contain regions with well-dispersed nanoparticles, predominantly exhibiting spherical morphology, minimal agglomeration, and a relatively uniform size distribution (Figure 9).

Nonetheless, although some regions show well-defined morphology, it is important to emphasize that this behavior does not represent the overall structure of the nanocomposites, which exhibit nanoparticle agglomeration and coalescence in other analyzed areas—except for the 2MNP/PLA sample (Figures S4–S8). Such agglomeration is expected in surfactant-free syntheses due to the absence of steric or electrostatic barriers [52].

In the 2MNP/PLA sample, no agglomerates were observed across the analyzed fields (Figure S4). Although the polymer–nanoparticle interface is not explicitly resolved, the high degree of dispersion suggests the presence of specific interactions between the PLA matrix and the magnetite nanoparticles. These interactions may involve hydrogen bonding, dipole–dipole attractions, coordination mechanisms, or physical anchoring, all of which can promote stabilization and prevent aggregation. With a mean particle diameter of approximately 2.8 nm and excellent dispersion, this formulation is particularly promising for applications such as controlled drug delivery, magnetic sensors, or functional nanomaterials.

In the 5MNP/PLA sample, a diffuse “halo” surrounding individual nanoparticles was observed (Figure S5b,c), likely corresponding to partial encapsulation by PLA. This feature reinforces the presence of polymer–particle interaction and may enhance colloidal stability and dispersion [10,16], in addition to the previously mentioned improvement in oxidation stability. Although a few larger agglomerates are present (Figure S5d,e), they are spatially isolated and do not dominate the sample morphology. The mean particle diameter lies between 3 and 4 nm, indicating good dispersion and integration into the polymer matrix.

The 10MNP/PLA composite exhibits a tendency toward particle growth, increased morphological irregularity, and the appearance of larger aggregates (Figure S6). Despite these changes, the majority of particles remain within the 4–5 nm range, suggesting that nucleation control is still effective, although morphological uniformity begins to decline.

The 15MNP/PLA and 20MNP/PLA samples appear to approach the magnetite loading threshold. In the 15MNP/PLA formulation (Figure S7) a notably higher nanoparticle density per area is observed, suggesting either partial phase separation or limited matrix accommodation. Particles remain in the 4–5 nm size range but are more densely packed, and large, irregular agglomerates are more frequent. In the sample containing 20% magnetite (20MNP/PLA), a pronounced deterioration in nanoparticle dispersion is observed, characterized by a significant increase in particle size and a marked reduction in the population of smaller nanoparticles (Figure S8). The size distribution shows average diameters ranging from approximately 5 to 15 nm, excluding visibly larger agglomerated structures. These findings indicate a loss of nucleation control and compromised dispersion efficiency at higher filler loadings.

While the 15% and 20% composites provide increased functional content due to the higher magnetite concentration, the loss of morphological homogeneity may compromise their mechanical, magnetic, and functional properties [53]. Although polymer–nanoparticle interactions may still occur, their effectiveness appears diminished at elevated loadings.

The TEM micrographs analyses support the hypothesis from the Raman analysis that PLA is coating the magnetite nanoparticles, which is consistent with our previous findings [25]. The polymer coating on magnetite is significant because it enhances the colloidal stability of the composite and reduces the aggregation tendency of the nanoparticles [10,16], in addition to the previously mentioned improvement in oxidation stability. The images that show some regions of nanoparticle aggregation are expected due to the absence of surfactants in the synthesis.

**Figure 10:**
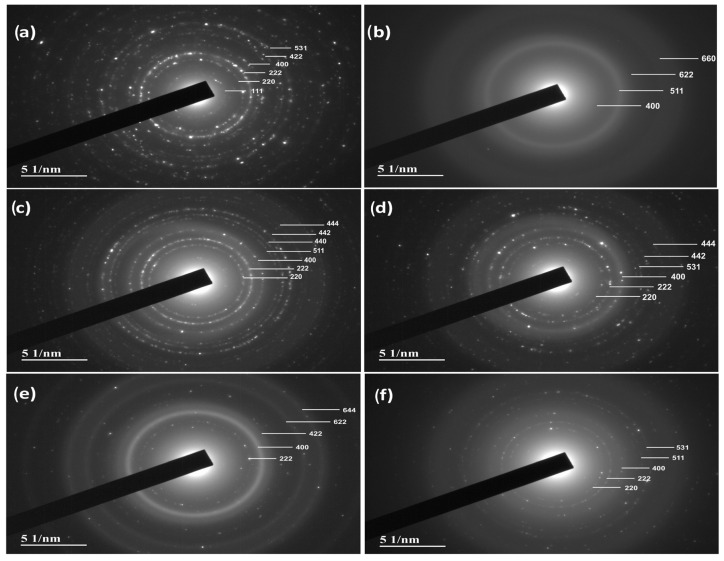
SAED images obtained from TEM of (**a**) MNP, (**b**) 2MNP/PLA, (**c**) 5MNP/PLA, (**d**) 10MNP/PLA, (**e**) 15MNP/PLA, and (**f**) 20MNP/PLA.

Figure 10 shows the selected area electron diffraction (SAED) or selected area diffraction pattern (SADP) images of the pure magnetite sample (a) and the MNP/PLA composites at concentrations of 2, 5, 10, 15, and 20% (b–f), respectively, while Table S1 presents the corresponding calculations. The SAED pattern of pure magnetite shows a series of diffraction rings corresponding to the crystal planes of the inverse spinel structure (cubic, Fd¯3m), with the (311) reflection—which is typically the most intense in XRD patterns—appearing weak or nearly absent, while the (220), (400), (511), and (440) reflections are clearly observed. This behavior may result from several factors, including the nanometer-scale size of the particles, sample thickness, crystallinity, particle orientation relative to the electron beam, and the specific area of the sample illuminated by the beam.

In the composite samples, the rings remain visible, but with significantly lower intensity and sharpness. This attenuation is primarily due to the low concentration of crystalline magnetite particles in the polymer matrix, which weakens the signal. Additionally, the small size of the particles—on the nanometer scale—causes their peaks to appear broad and weak, making them easily overlapped by the amorphous background of the PLA. This effect is particularly evident in the (311) plane structure, which, although intense in pure magnetite, becomes almost invisible in the composite samples. The dispersion of the particles within the polymer matrix, combined with the nanometric size of the crystallites, contributes to this detection challenge—a common feature in composites with inorganic particles dispersed in organic matrices. Therefore, the SAED results align with the microstructure observed in the TEM images: nanometric magnetite particles are relatively well dispersed in the polymer matrix at low concentrations, leading to low-intensity, broad diffraction peaks.

### 3.8. Textural Properties of xMNP/PLA Nanocomposites

The textural characteristics of a material—such as specific surface area, pore volume, and pore distribution—significantly influence its structure and reactivity [54]. Therefore, some of these features were analyzed in the synthesized nanocomposites (Table 4 and Figure S9). The synthesized magnetite (with a crystal size of approximately 20 nm) exhibited similar textural properties to those previously reported in the literature [37,55]. The nitrogen adsorption isotherms of pure MNPs primarily correspond to a type IV(a) isotherm with H1-type hysteresis, according to the IUPAC classification [56]. The MNP displays a predominantly mesoporous structure, with a specific surface area of 41.5 m^2^/g, approximately 25% of which is contributed by micropores. From the total pore volume (0.15 cm^3^/g), only about 3% is attributed to micropores (Table 4).

The synthesized PLA likely exhibits a mixture of type II and type IV(a) isotherms with H3-type hysteresis, as the adsorption branch loop resembles that of a type II isotherm. This kind of loop is typically associated with non-rigid aggregates of plate-like particles and the presence of large pores (macropores) [56,57]. The extremely low specific surface area (0.4 m^2^/g), absence of detectable micropores, and very low total pore volume (0.0006 cm^3^/g) confirm that this material is essentially nonporous (Table 4).

The nanocomposites exhibit isothermal profiles with very low N_2_ adsorption capacity, resulting in low specific surface areas (averaging ~2.1 m^2^/g). The isotherms resemble type II behavior, typical of nitrogen physisorption on nonporous or macroporous adsorbents [56]. The slight curvature and the absence of a well-defined “point B” on the isotherm indicate a notable overlap between monolayer coverage and the onset of multilayer adsorption. The presence of a hysteresis loop also imparts characteristics of a type IV(a) isotherm with H3-type hysteresis, related to pore networks formed by macropores that are not completely filled with condensate. Additionally, the average total pore volume increased to 0.03 cm^3^/g, compared to just 0.0006 cm^3^/g for pure PLA, which can be attributed to the incorporation of MNPs into the composites. This is corroborated by findings from other PLA composite fillers [58,59].

It is worth mentioning that the incorporation of magnetite nanoparticles, which have a high specific surface area when isolated (~42 m^2^/g), into the dense PLA matrix (0.4 m^2^/g) resulted in only a modest increase in the total surface area of all composites, reaching a maximum of 2.3 m^2^/g. Considering all major textural parameters described, this slight increase confirms that only a small fraction of the MNP surface remains accessible, indicating successful partial dispersion in the matrix. This behavior is common in polymeric nanocomposites, especially when the matrix is low in porosity and the reinforcement is highly porous [58,59]. The significant reduction in the accessible surface area of the MNPs can be attributed to factors such as partial encapsulation of the nanoparticles by polymer chains, pore blockage, and the occurrence of particle agglomeration or coalescence, as also observed in the TEM micrographs. These phenomena limit the exposure of the reinforcement surface to the adsorbate gas during measurements, thereby reducing its contribution to the composite’s total surface area.

### 3.9. Magnetic Properties of xMNP/PLA Nanocomposites

**Figure 11:**
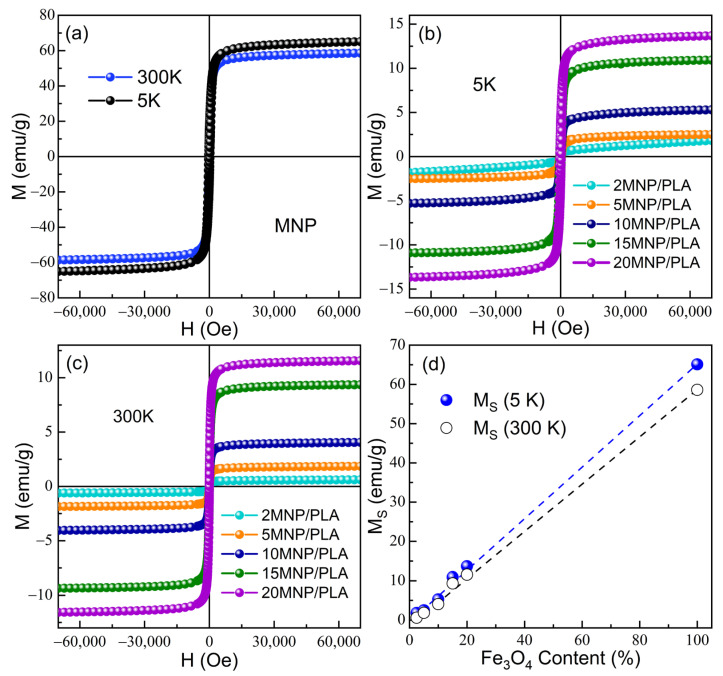
Magnetization curves (M) as a function of the magnetic field (H) performed at 300 K and 5 K for (**a**) MNP pure; (**b**) 5 K for xMNP/PLA nanocomposites; (**c**) 300 K for xMNP/PLA nanocomposites; and (**d**) the saturation magnetization as a function of MNP concentration.

The magnetization (M) versus applied magnetic field (H) for pure Fe_3_O_4_ magnetic nanoparticles (MNPs), measured at 300 K and 5 K, is presented in Figure 11a. The saturation magnetization (Ms) values are 65.1 emu/g at 5 K and 58.6 emu/g at 300 K. These values are lower than those of bulk magnetite, which is ~90 emu/g [60]. These results strongly suggest the formation of a maghemite phase and the occurrence of magnetic disorder at the particle surface, which leads to the saturation magnetization reduction, as corroborated by Raman and XRD data analysis, showing a lattice constant consistent with a maghemite phase and small-sized particles [61,62]. Moreover, the hysteresis loops show a coercive field of 338 Oe at 5 K, which decreases to 108 Oe at 300 K as the temperature increases. With the incorporation of MNPs into PLA, the magnetic properties progressively change, as reflected in the magnetization curves displayed in Figure 11b,c. The magnetization curves for all nanocomposites exhibit an increasing trend in Ms with increasing Fe_3_O_4_ content, as clearly illustrated in Figure 11d. For instance, the sample with 2.0% MNP shows a very low Ms of 1.8 emu/g at 5 K and 0.6 emu/g at 300 K, while that for 20% MNP rises to 13.7 emu/g at 5 K and 11.6 emu/g at 300 K, reflecting the proportional contribution of the magnetic content in the nanocomposites (Table 5). A particularly interesting observation emerges in the 2MNP/PLA sample, where the coercive field at 300 K is zero. This strongly suggests that the nanoparticles are in a superparamagnetic-like regime, where thermal energy overcomes the magnetic anisotropy barrier, leading to zero remanence and coercive field. This behavior is supported by the reduction in crystallite size observed via XRD (see inset of Figure 1d), which indicates that at low concentrations, the particle size is smaller (below ~20 nm) and dispersed well, resulting in superparamagnetic-like behavior at high temperatures. In contrast, as the MNP content increases, coercivity values grow significantly: for example, 5% MNP/PLA exhibits Hc = 38 Oe at 300 K, while 15% MNP/PLA reaches 78 Oe, indicating a clear shift toward stable ferrimagnetic behavior at room temperature, likely due to the increase in particle size and interparticle interactions caused by higher concentrations. This trend is even more pronounced at low temperature (5 K), where thermal agitation is suppressed. The coercive field increases from 237 Oe (2.0% MNP/PLA) to over 400 Oe for the 5%, 15%, and 20% composites. Such behavior must be related to particle size growth and the enhancement of particles’ interaction at higher MNP concentrations due to the increase in MNP loading.

**Figure 12:**
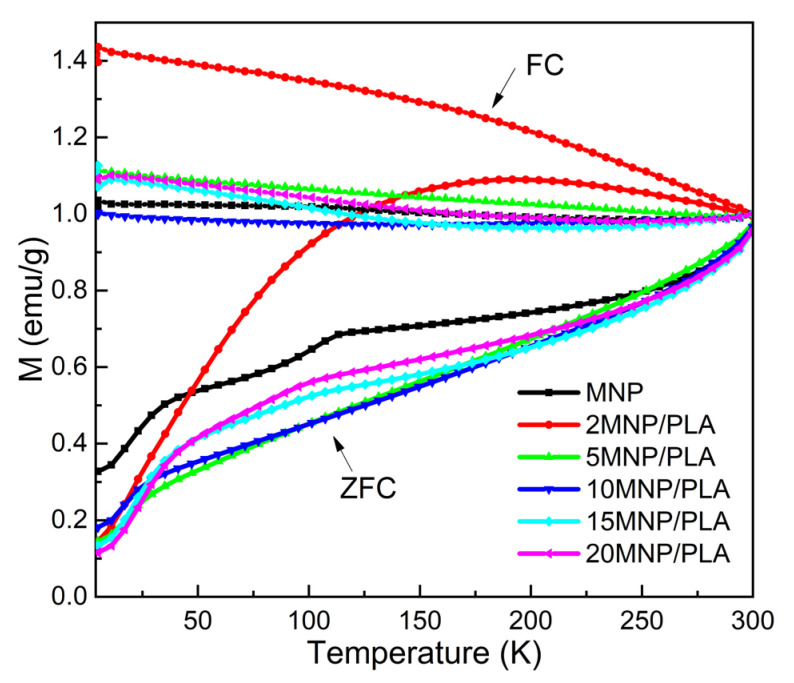
ZFC and FC magnetization curves measured with a magnetic field of 30 Oe for the xMNP/PLA Nanocomposites.

Figure 12 shows the zero-field-cooled (ZFC) and field-cooled (FC) magnetization curves for pure MNPs and MNP/PLA nanocomposites, measured as a function of temperature. These measurements provide insights into the thermal magnetic behavior and the degree of magnetic interactions between particles. A distinct behavior is observed for the sample with 2.0 wt.% MNPs. This sample shows a well-defined maximum in the ZFC curve at approximately 200 K, which is indicative of a blocking temperature (TB) typical of superparamagnetic behavior in weakly interacting particles and is consistent with the zero coercive field determined at 300 K. In contrast, for nanocomposites with higher MNP concentrations (≥5 wt.%), the ZFC curves do not exhibit a clearly defined maximum. Instead, irreversibility in the ZFC and FC curves persists even near 300 K, which agrees with the results obtained from M vs. H curves and likely suggests the occurrence of strong particle–particle interactions in samples with higher MNP concentrations.

## 4. Conclusions

Composites containing magnetic nanoparticles (MNPs) have been the focus of growing research due to their potential applications in biomedicine. In this study, we prepared MNP/PLA nanocomposites with varying loadings (2, 5, 10, 15, and 20 wt.%) using a sonochemical synthesis method. A range of techniques were employed to investigate their structures and properties, including XRD, FT-IR, FT-Raman, SEM, TEM, TG, and DTA, as well as textural (specific surface area and porosity) and magnetic analyses.

The aqueous sonochemical synthesis proved to be an effective method for preparing MNP/PLA nanocomposites, with less than 2% deviation from the theoretical MNP loadings. A small presence of maghemite in the composites was detected by XRD patterns, FT-Raman, and magnetic measurements. Chemical and electrostatic interactions between magnetite and PLA were indicated by FT-IR analysis, supported by a higher transition temperature (145 °C) compared to pure PLA (139 °C), as observed in TG/DTG and DTA. The MNP were well-dispersed in the PLA matrix, with minimal agglomeration and relatively uniform size distribution, as confirmed by SEM and TEM. The coating of the MNP with PLA protected the nanoparticles from oxidation, improving their stability. To the best of our knowledge, this is the first study to investigate the textural properties of these composites using N_2_ adsorption. The isotherms further supported the coating of the MNP by PLA, with the polymer blocking the original pores of magnetite, resulting in a low N_2_ adsorption capacity for the nanocomposites. Moreover, magnetic analyses of all composites revealed superparamagnetic-like behavior for the 2 wt.% MNP/PLA sample at 300 K, while the higher loading composites exhibited stable ferrimagnetic behavior. It is expected that the newly characterized properties of these nanocomposites could aid in decision-making regarding their applicability in biomedical areas, such as drug delivery or PLA-based bone scaffolds and screws.

## Figures and Tables

**Table 1:** Inorganic elemental content (Fe_3_O_4_) of the composite samples.

Material	Fe_3_O_4_ Content (%)	Difference (%) ^a^
2MNP/PLA	2.0	−0.5
5MNP/PLA	4.8	−0.2
10MNP/PLA	8.3	−1.7
15MNP/PLA	13.1	−1.9
20MNP/PLA	18.2	−1.8

^a^ The difference is related to the theoretical value (2.5; 5; 10; 15; and 20 wt.%).

**Table 2:** Temperature of maximum degradation (T_1_ and T_2_) of the composite samples. The errors associated with the temperatures are ±1 °C.

Material	T_1_ (°C)	T_2_ (°C)
2MNP/PLA	281	361
5MNP/PLA	276	371
10MNP/PLA	276	361
15MNP/PLA	275	334
20MNP/PLA	271	336

**Table 3:** Temperature values of T_PT_, T_TD_, T_OD1_, and T_OD2_ of PLA and composite samples. The errors associated with the temperatures are ±1 °C.

Material	T_PT_ (°C) ^a^	T_TD_ (°C) ^b^	T_OD1_ (°C) ^c^	T_OD2_ (°C) ^c^
PLA	139	305	338	366
2MNP/PLA	145	276	303	370
5MNP/PLA	143	269	294	381
10MNP/PLA	143	270	293	365
15MNP/PLA	143	269	311	331
20MNP/PLA	145	259	318	350

^a^ Temperature of phase transition (T_PT_). ^b^ Temperature of thermal degradation (T_TD_). ^c^ Temperature of oxidative degradation (T_OD1_ and T_OD2_).

**Table 4:** Some textural properties of MNP, PLA, and nanocomposites. The errors associated with the surface areas and volumes are about 5%.

Material	S_BET_ ^a^ (m^2^/g)	S_Micro_ ^b^ (m^2^/g)	V_p_ ^c^ (cm^3^/g)	V_Micro_ ^d^ (cm^3^/g)
MNP	41.5	10.7	0.15	0.0047
PLA	0.4	0	0	0
2MNP/PLA	2.2	1.4	0.03	0.0004
5MNP/PLA	2.3	1.4	0.04	0.0005
10MNP/PLA	1.6	1.3	0.02	0.0005
15MNP/PLA	2.0	1.4	0.02	0.0002
20MNP/PLA	2.1	2.2	0.04	0.0008

^a^ Specific surface area obtained by the BET method in the P/P_0_ range of 0.01 to 0.1. ^b^ Microporous surface area obtained by the t-plot method. ^c^ Total pore volume calculated by the quantity of gas adsorbed at P/P_0_ = 0.98. ^d^ Micropore volume calculated by t-plot method.

**Table 5:** Parameters obtained from the analysis of the magnetization curves for the composite samples.

Material	H_C_ (5 K)	H_C_ (300 K)	M_S_ (5 K)	M_S_ (300 K)
MNP	339	108	65.1	58.6
2MNP/PLA	237	0	1.8	0.6
5MNP/PLA	411	38	2.5	1.9
10MNP/PLA	313	46	5.3	4.1
15MNP/PLA	403	78	10.9	9.4
20MNP/PLA	403	59	13.7	11.6

## Data Availability

All data are within the article and the Supplementary Materials and may be available upon request.

## Supplementary Materials

The following supporting information can be downloaded at: https://www.mdpi.com/article/10.3390/polym17121713/s1: Figure S1. (a) ^1^H and (b) ^13^C NMR spectra of PLA sample; Figure S2. XRD pattern of PLA and magnetite MNP samples; Figure S3. EDX elemental analysis obtained from SEM images of: (a) 5MNP/PLA, (b) 10MNP/PLA, (c) 15MNP/PLA, and (d) 20MNP/PLA samples. Copper peaks are from the sample preparation; Figure S4. TEM images of the 2MNP/PLA composite at different magnifications: (a) 5 nm, (b) 20 nm, (c) 50 nm, (d) 100 nm, and (e) 500 nm; Figure S5. TEM images of the 5MNP/PLA composite at different magnifications: (a) 5 nm, (b) 50 nm, (c) 100 nm, (d) 200 nm, and (e) 500 nm; Figure S6. TEM images of the 10MNP/PLA composite at different magnifications: (a) 5 nm, (b) 20 nm, (c) 50 nm, (d) 100 nm, and (e) 200 nm; Figure S7. TEM images of the 15MNP/PLA composite at different magnifications: (a) 5 nm, (b) 50 nm, (c) 100 nm, (d) 200 nm, and (e) 500 nm; Figure S8. TEM images of the 20MNP/PLA composite at different magnifications: (a) 5 nm, (b) 50 nm, (c) 100 nm, (d) 200 nm, and (e) 500 nm; Table S1. Comparative distances (*d*) between atoms in the (h–l) planes obtained from SAED images and those from the crystallographic pattern of magnetite (COD database, ID 9006189). Figure S9. Nitrogen adsorption–desorption isotherms at −196 °C for: (a) MNP, (b) PLA, and (c) 5MNP/PLA.
