# Supplementary material for: Tailoring the Properties of Magnetite/PLA Nanocomposites: A Composition-Dependent Study
Source: Polymers (Basel). 2025 Jun 19;17(12):1713. doi: 10.3390/polym17121713 (PMC12196626; doi:10.3390/polym17121713)
Supplement: Supplementary file 1 [file polymers-17-01713-s001.zip › polymers-3710156-supplementary.pdf]

Supplementary Material

Tailoring the Properties of Magnetite/PLA Nanocomposites: A Composition-Dependent Study

by

Mariana Martins de Melo Barbosa [a*], Juliene Oliveira Campos de França [a],

Quezia dos Santos Lima [a], Sílvia Cláudia Loureiro Dias [a], Carlos A. Vilca Huayhua [b],

Fermín F. H. Aragón [b,c], J. A. H. Coaquira [b*], and José Alves Dias [a*]

1. Characterization Methods

1.1 Proton and Carbon-13 Nuclear Magnetic Resonance (^{1}H and ^{13}C NMR)

The proton and carbon-13 nuclear magnetic resonance (^{1}H and ^{13}C NMR) were obtained for PLA on $CDCl_3$ on a spectrometer (Bruker, Avance III HD-Ascend model, at 14.1 T, 600 MHz, Ettlingen, Germany). The acquisition conditions were a single pulse of 4.5 μs duration, acquisition time of 0.1 s, interval between pulses of 1 s, minimum of 20 acquisitions, and internal reference of TMS (δ = 0.0 ppm).

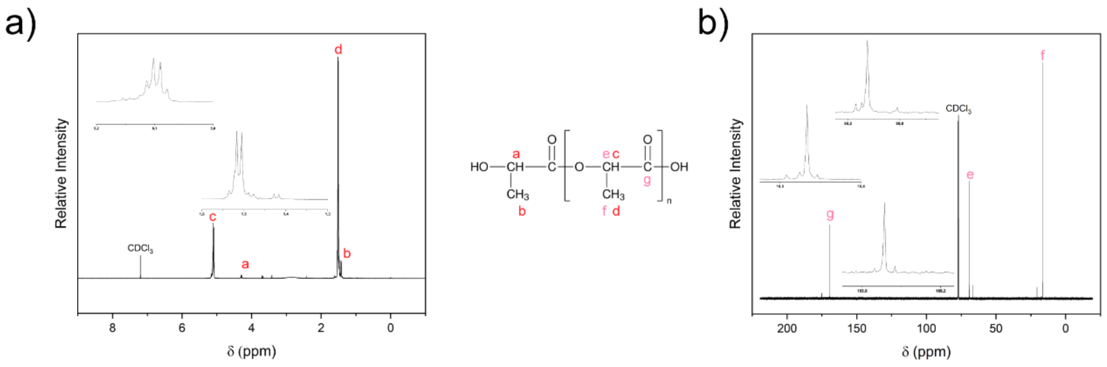

Figure S1. a) ^{1}H and b) ^{13}C NMR spectra of PLA sample.

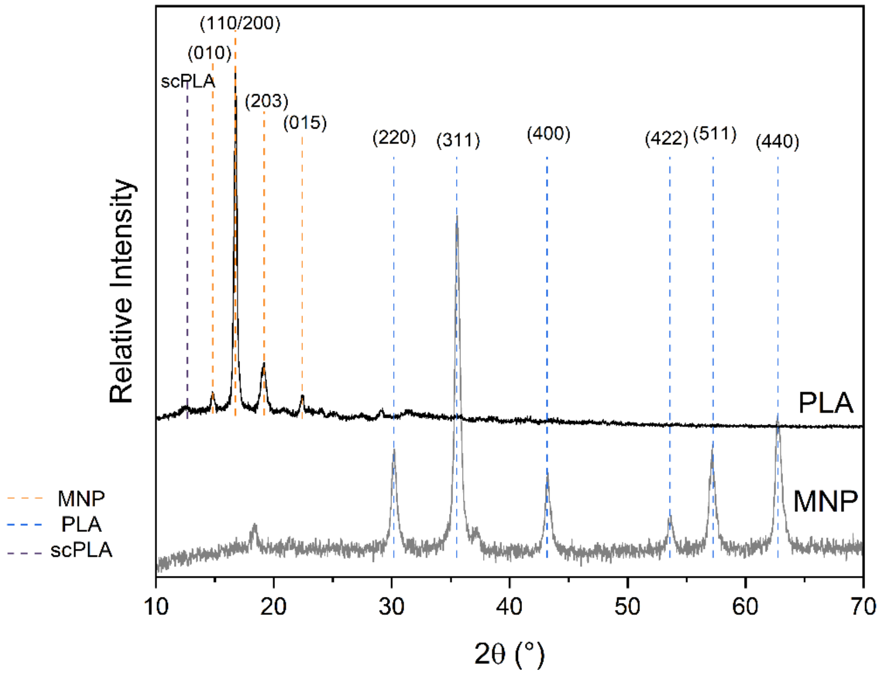

Figure S2. XRD pattern of PLA and magnetite MNP samples.

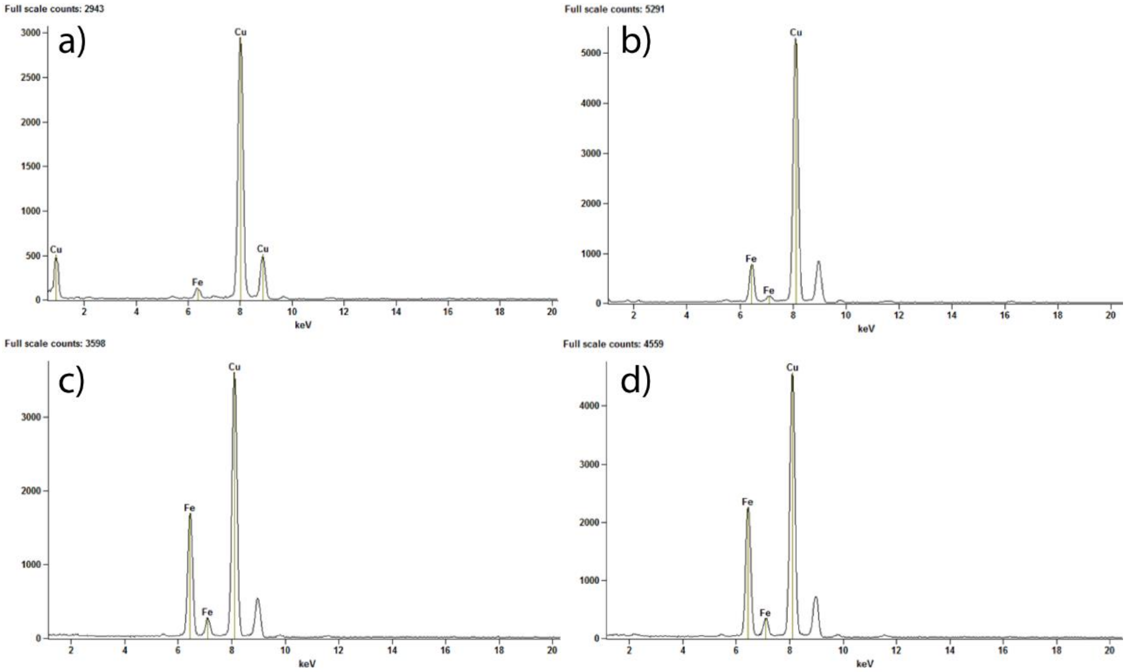

Figure S3. EDX elemental analysis obtained from SEM images of: a) 5MNP/PLA, b) 10MNP/PLA, c) 15MNP/PLA, and d) 20MNP/PLA samples. Copper peaks are from the sample preparation.

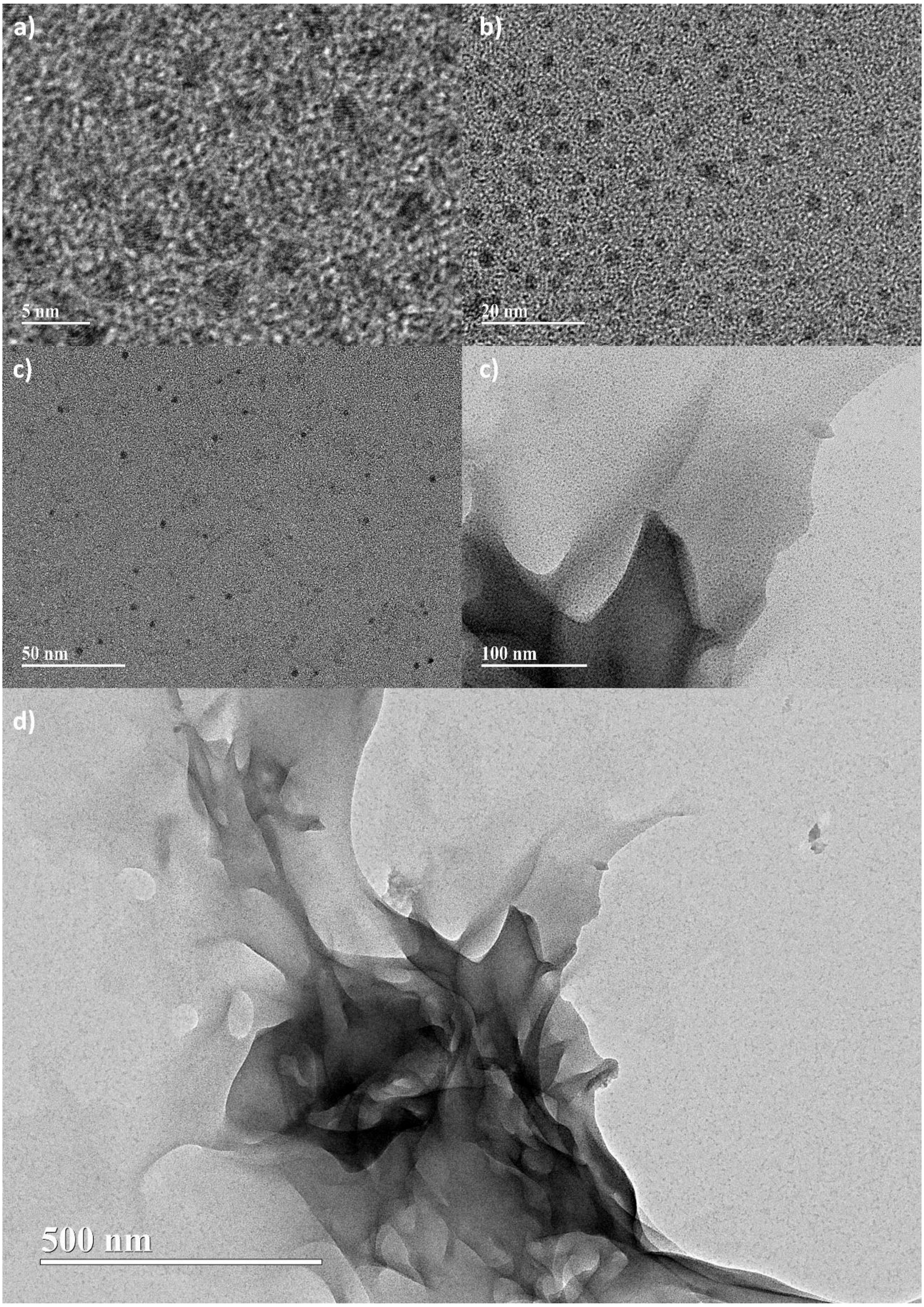

Figure S4. TEM images of the 2MNP/PLA composite at different magnifications: (a) 5 nm, (b) 20 nm, (c) 50 nm, (d) 100 nm, and (e) 500 nm.

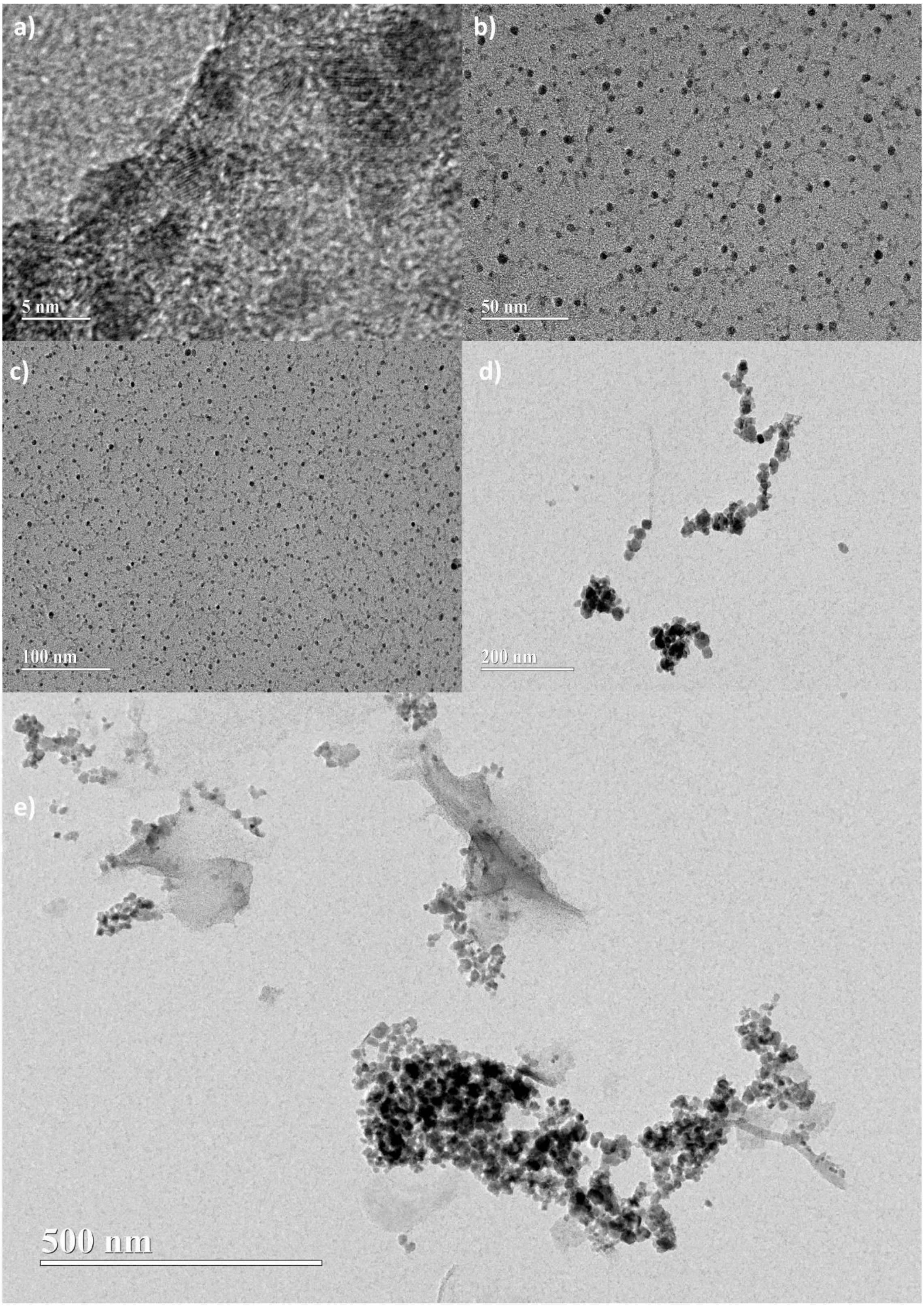

Figure S5. TEM images of the 5MNP/PLA composite at different magnifications: (a) 5 nm, (b) 50 nm, (c) 100 nm, (d) 200 nm, and (e) 500 nm.

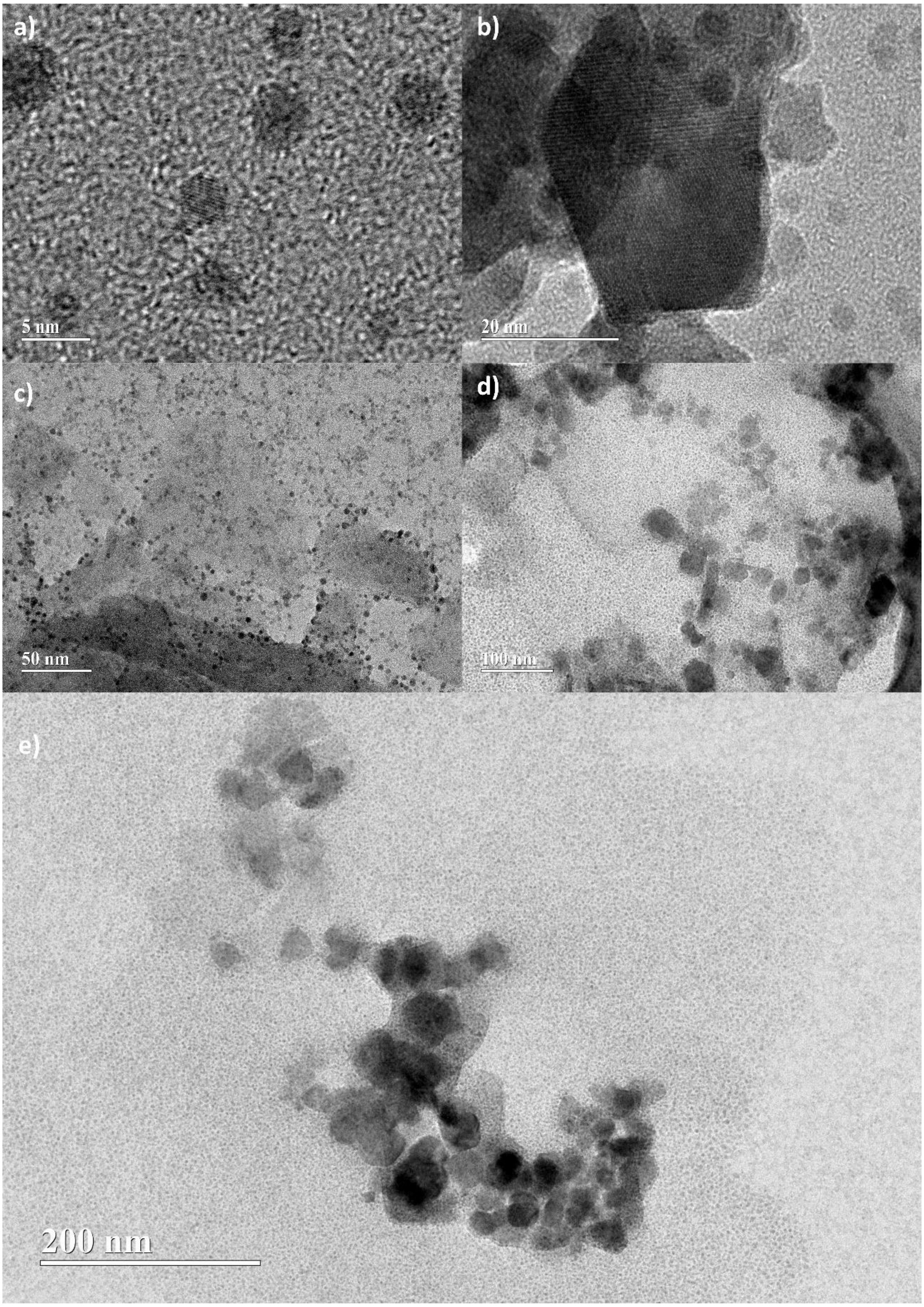

Figure S6. TEM images of the 10MNP/PLA composite at different magnifications: (a) 5 nm, (b) 20 nm, (c) 50 nm, (d) 100 nm, and (e) 200 nm.

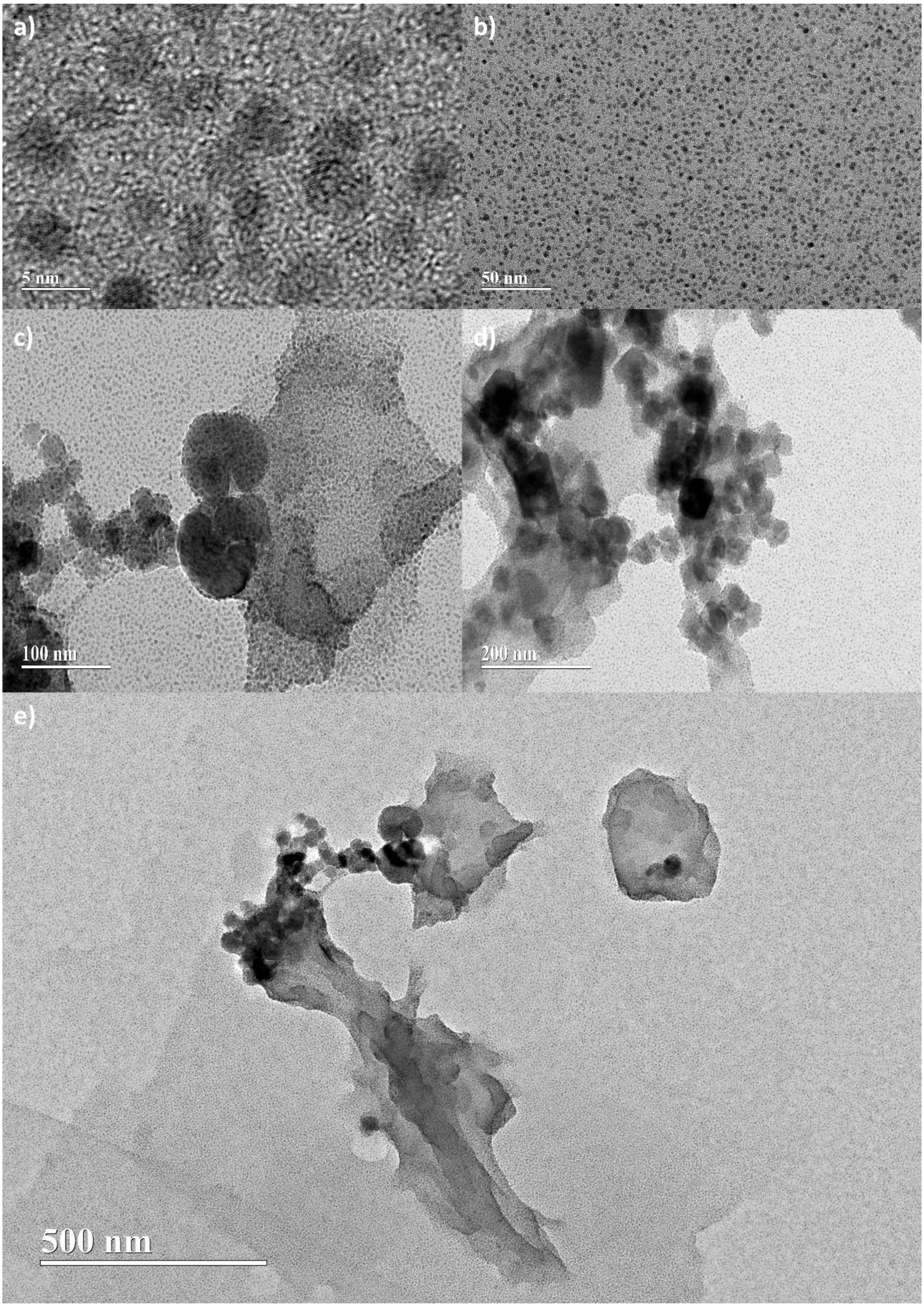

Figure S7. TEM images of the 15MNP/PLA composite at different magnifications: (a) 5 nm, (b) 50 nm, (c) 100 nm, (d) 200 nm, and (e) 500 nm.

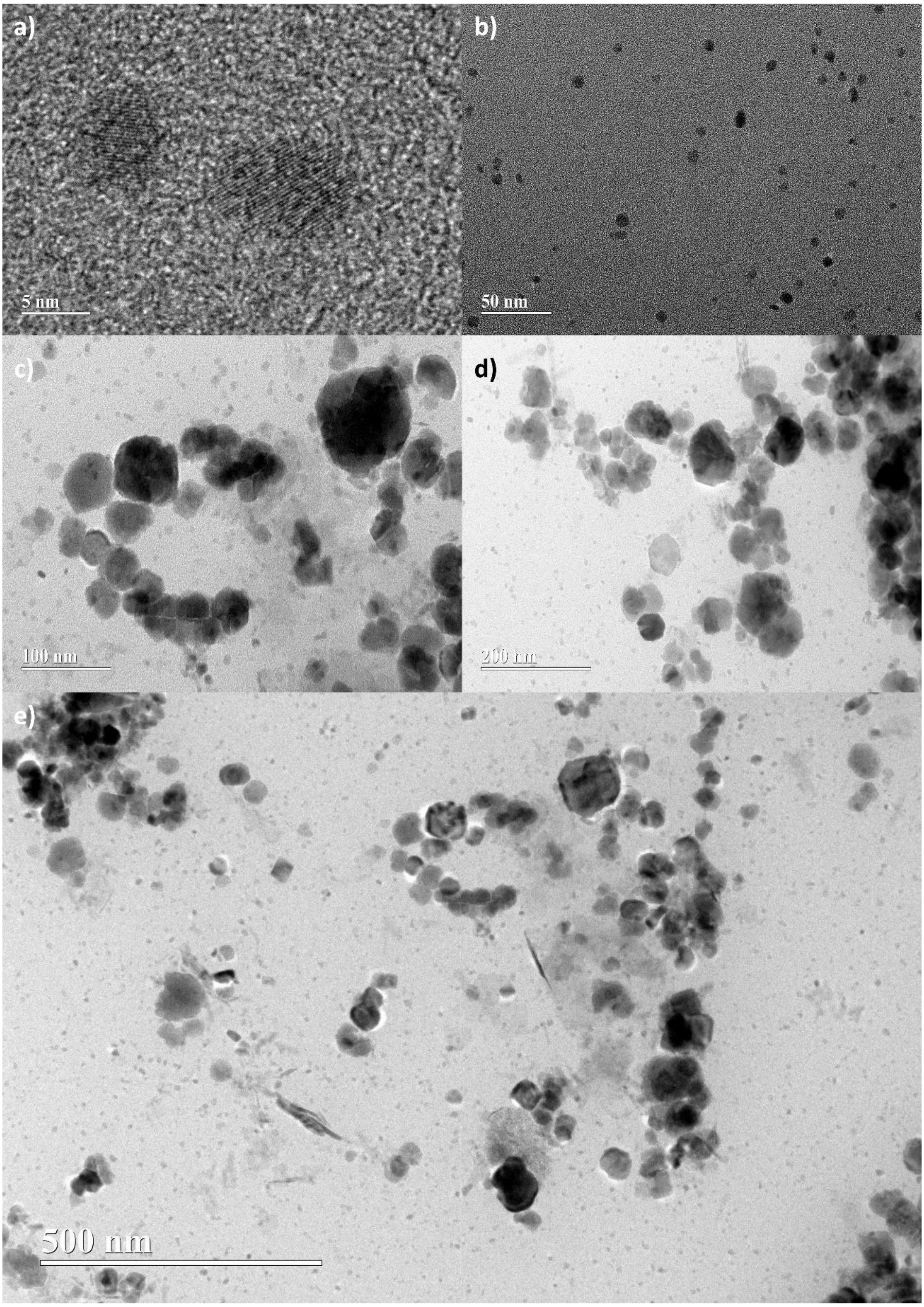

Figure S8. TEM images of the 20MNP/PLA composite at different magnifications: (a) 5 nm, (b) 50 nm, (c) 100 nm, (d) 200 nm, and (e) 500 nm.

Table S1. Comparative distances (d) between atoms in the (h, k, l) planes obtained from SAED images and those from the crystallographic pattern of magnetite (COD database, ID 9006189).

Sample	d_{EXP} (Å) [a]	d_{CIF} (Å) [b]	Index (h, k, l) [c]
MNP	4.774	4.848	111
	2.870	2.969	220
	2.417	2.424	222
	2.017	2.099	400
	1.667	1.714	422
	1.424	1.419	531
2MNP/PLA	2.097	2.099	400
	1.592	1.616	511
	1.260	1.266	622
	0.971	0.990	660
5MNP/PLA	2.822	2.969	220
	2.413	2.424	222
	1.995	2.099	400
	1.633	1.616	511
	1.530	1.588	440
	1.406	1.400	442
	1.226	1.212	444
10MNP/PLA	2.817	2.969	220
	2.418	2.424	222
	1.991	2.099	400
	1.421	1.419	531
	1.408	1.400	442
	1.220	1.212	444
15MNP/PLA	2.836	2.969	220
	2.439	2.424	222
	2.020	2.099	400
	1.727	1.714	422
	1.232	1.212	444
20MNP/PLA	2.873	2.969	220
	2.422	2.424	222
	2.022	2.099	400
	1.556	1.616	511
	1.417	1.419	531

[a] The distance between two atomic planes (h, k, l) obtained from SAED images.

[b] The distance between two atomic planes (h, k, l) obtained from magnetite in the COD database (ID 9006189).

[c] Miller indices (h, k, l) obtained from the magnetite COD database (ID 9006189).

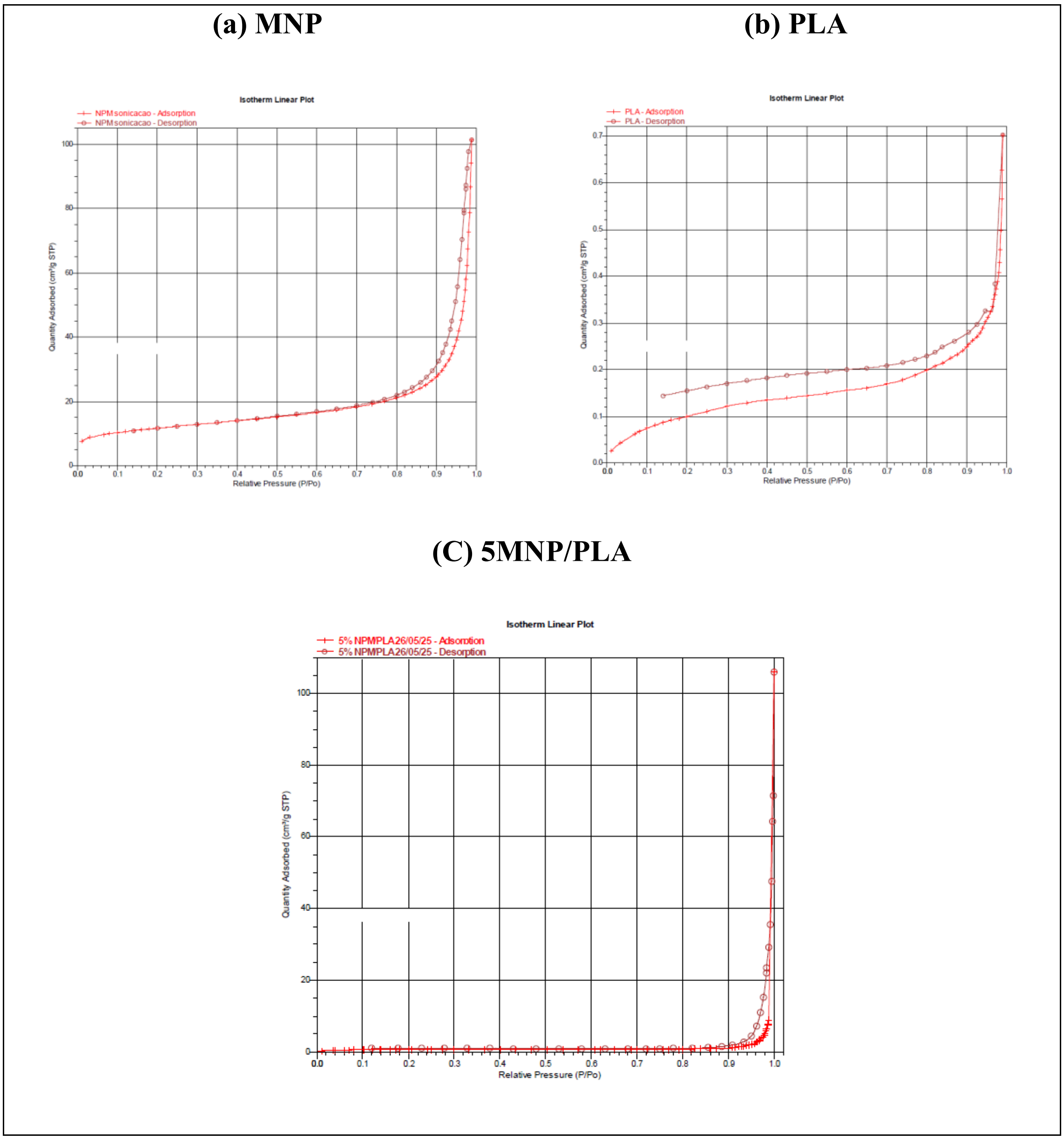

Figure S9. Nitrogen adsorption–desorption isotherms at -196 °C for: (a) MNP, (b) PLA, and (c) 5MNP/PLA.
